# Epigenetic Modifications of the *α*-Synuclein Gene and Relative Protein Content Are Affected by Ageing and Physical Exercise in Blood from Healthy Subjects

**DOI:** 10.1155/2018/3740345

**Published:** 2018-04-15

**Authors:** Simona Daniele, Barbara Costa, Deborah Pietrobono, Chiara Giacomelli, Caterina Iofrida, Maria Letizia Trincavelli, Jonathan Fusi, Ferdinando Franzoni, Claudia Martini

**Affiliations:** ^1^Department of Pharmacy, University of Pisa, 56126 Pisa, Italy; ^2^Department of Clinical and Experimental Medicine, University of Pisa, 56120 Pisa, Italy

## Abstract

Epigenetic regulation may contribute to the beneficial effects of physical activity against age-related neurodegeneration. For example, epigenetic alterations of the gene encoding for *α*-synuclein (*SNCA*) have been widely explored in both brain and peripheral tissues of Parkinson's disease samples. However, no data are currently available about the effects of physical exercise on *SNCA* epigenetic regulation in ageing healthy subjects. The present paper explored whether, in healthy individuals, age and physical activity are related to blood intron1-*SNCA* (*SNCA_I1_*) methylation, as well as further parameters linked to such epigenetic modification (total, oligomeric *α*-synuclein and DNA methyltransferase concentrations in the blood). Here, the *SNCA_I1_* methylation status increased with ageing, and consistent with this result, low *α*-synuclein levels were found in the blood. The direct relationship between *SNCA_I1_* methylation and *α*-synuclein levels was observed in samples characterized by blood *α*-synuclein concentrations of 76.3 ng/mg protein or lower (confidence interval (CI) = 95%). In this selected population, higher physical activity reduced the total and oligomeric *α*-synuclein levels. Taken together, our data shed light on ageing- and physical exercise-induced changes on the *SNCA* methylation status and protein levels of *α*-synuclein.

## 1. Introduction

Ageing is characterized by common cellular features, such as increased oxidative stress, reduction in protein synthesis, mitochondrial dysfunction, stem cell depletion, and telomere attrition [1]. Moreover, the ageing process is strictly linked to epigenetic control of the genome through DNA and histone modifications [1, 2], such that DNA methylation has been suggested as an ageing “clock” [3, 4].

In the central nervous system, DNA methylation [5] regulates memory formation [6] and its age-related disruption, which precede cognitive decline [7, 8]. The epigenome is extremely dynamic, with changes in response not only to ageing or development, but it is also affected by exogenous factors, such as nutrient availability and physical exercise [9–11]. Indeed, several studies indicate that epigenetic regulation may contribute to the widely known beneficial effects of regular exercise, although the exact mechanisms remain to be determined at molecular levels [12]. For example, regular exercise has been suggested to affect the methylation status of global and CpG-rich specific genes in the muscle cells, the different peripheral tissues, and the brain. Moreover, the gene for neurotrophic factor BDNF (brain-derived growth factor) is known to be remodelled epigenetically in the hippocampus in response to physical exercise [13]. Such studies are consistent with the hypothesis that epigenetics is central to coordinating the transcriptional response to the environment, as well as being involved in neuronal development, maintenance, and degeneration processes [14–16]. Based on this, it is not surprising that physical exercise has been suggested as a potential preventive countermeasure against many chronic and degenerative diseases [11, 17–19], including age-related neurodegeneration [20]. Among these, evidence was reported for Parkinson's disease (PD) [21–28], which is characterized by an accumulation of misfolded *α*-synuclein (*α*-syn) in Lewy bodies, causing synaptic dysfunction and neuronal loss [29–31]. The exercise-mediated neuroprotective effects against PD are related to a reduction in cerebral inflammation and protein misfolding, as well as changes in gene expression profiles [32, 33]. In this respect, in addition to *α*-syn misfolding, epigenetic regulation of its encoding gene (*SNCA*) has been widely explored, particularly focusing on the methylation status of the intron1 CpG island [34–37]. Indeed, this intron1 region was demonstrated to control gene expression by the differential methylation of its CpG island, as well as by the recruitment of different transcription factors [34, 35, 38]. Thus, a decrease of intron1 methylation of *SNCA* has been hypothesized to increase *α*-syn expression in brain tissues and to lead to PD pathogenesis factors [34, 35]. Moreover, the pathological significance of DNA methylation was also highlighted in peripheral tissues. For example, leukocytes from PD subjects showed hypomethylation of intron1-*SNCA* compared to controls. [36, 39, 40]. By contrast, other studies reported no difference between PD and healthy subjects [41, 42].

In the light of the emerging role of physical activity in the management of neurodegenerative diseases, gaining further insight into epigenetic mechanisms regulating gene transcription during exercise will help further improve lifestyle interventions. Therefore, the aim of our study was to investigate the potential impact of regular training on *α*-syn expression and specific DNA methylation in healthy subjects. In particular, sedentary and exercise subjects were enrolled to determine the *α*-syn methylation status of intron1 CpG islands. The methylation degrees were correlated with the amount of total and oligomeric *α*-syn content in red blood cells (RBCs), selected as a valid cellular model, because they accumulate *α*-syn and are particularly susceptible to oxidative stress [43–45].

## 2. Methods

### 2.1. Study Population

Thirty-two endurance athletes (ATHL, mean age 41.4 ± 13.7 years) and 52 healthy sedentary controls (SED, mean age 45.9 ± 14.3 years) were selected for the study (Table 1). Only subjects free of cardiovascular diseases were included. Major inclusion criteria were reported previously [46]. All subjects were nonsmoking and had no regular medication or supplementation of vitamins or trace elements. Moreover, subjects with a family history of cardiovascular disease, hypertension, and other cardiovascular risk factors were excluded [46, 47].

Athletes were recruited from the outpatient clinic of the Sports Medicine Unit of the Department of Clinical and Experimental Medicine of the University of Pisa. Sedentary subjects were not performing any regular physical training.

The level of intensity was evaluated by the use of the 15-point Borg RPE scale [46, 48, 49] for each participant. The study population was further divided into the following subgroups: (1) sedentary (SED, *n* = 52) and athlete (ATHL, *n* = 32) subgroups, when the parameter for subdivision was the degree of physical activity; (2) “young” (*n* = 50) and “older” (*n* = 34) subgroups, when the parameter for subdivision was age.

This study was approved by the Ethics Committee of the Great North West Area of Tuscany (271/2014 to F. F.) and was performed in accordance with the Declaration of Helsinki. All subjects gave informed and written consent to participate in the study [46].

### 2.2. RBC Collection

Whole blood was collected into EDTA tubes. RBCs were separated from plasma by centrifugation at 200 ×g at 4°C for 10 min [46]. The resulting pellet was washed three times with PBS and frozen at −20°C until use. For athletes, the time period between the last exercise session and blood sampling was at least 48 h. The various parameters estimated included (i) CpG site methylation within *SNCA* intron1 (*SNCA_I1_*); (ii) concentration of the protein encoded by *SNCA* (*α*-syn), considering total levels and the contribution of its oligomeric form; and (iii) levels of the DNA methyltransferase enzymes of maintenance (Dnmt1) and *ex novo* (Dnmt3a).

### 2.3. *SNCA* Intron1 Relative DNA Methylation Analysis

Genomic DNA was extracted from the whole blood of healthy individuals (*n* = 84) using the QIAamp DNA Blood Kit (catalogue number: 51104; QIAGEN, CA, USA) and quantified using a NanoDrop Lite Spectrophotometer (Thermo Fisher Scientific Inc., USA). The *SNCA* intron1 region previously associated with low levels of CpG island methylation in PD patients [35–37, 40, 50] was considered in the present study. Intron1 methylation levels were assessed by methylation-sensitive restriction enzyme (MRSE) digestion of genomic DNA followed by quantitative real-time polymerase chain reaction (PCR) according to Pihlstrøm and coworkers [36]. Briefly, for each DNA sample (20 ng), duplicates of a “test reaction” (with the MRSEs AccII and HpaII) and a “reference reaction” (without MSREs) were incubated at 37°C for 2 h. Then, samples were amplified using forward and reverse primers (FOR-5′ATTAGGCTGCTTCTCCGGGATC-3′, REV-5′GTTCTCAGCCTCCACCCTAG-3′). A melt curve was performed at the end of the experiment to confirm amplification specificity. The proportion of genomic DNA methylated at cut sites was calculated with the equation percent methylation = 100 × 2^−(CT[test reaction] − CT[reference reaction])^.

### 2.4. Levels of DNA Methyltransferase (Dnmt)

The concentrations of Dnmt1 and Dnmt3a were determined in the blood by specific immunoenzymatic assays, following the manufacturer's instructions (Biomatik Corporation, Ontario, Canada, http://www.biomatik.com). Briefly, standards or samples were added to the appropriate precoated microtitre plate wells with a biotin-conjugated antibody. Then, samples were incubated with avidin-HRP followed by the addition of the TMB substrate solution (3,3′,5,5′-tetramethylbenzidine). After stopping the reaction, the absorbance was read at 450 nm (http://www.biomatik.com).

### 2.5. Detection of Total *α*-Synuclein

Total *α*-syn was measured in RBCs as previously described [46, 51]. In brief, precoated wells (*α*-syn full-length antibody, sc-10717, Santa Cruz Biotechnology) were treated with bovine serum albumin (BSA). RBCs (0.150 mg/100 *μ*l) were captured on wells for 2 h at 25°C, and aliquots of recombinant *α*-syn were analysed in parallel to obtain a standard curve. After extensive washing, the samples were treated with a mouse monoclonal antibody to *α*-syn (sc-12767, Santa Cruz Biotechnology) and subsequently with an antimouse HRP antibody [46]. The samples were washed three times with PBS-T (phosphate-buffered saline containing 0.01% Tween 20) before the addition of the enzyme substrate TMB (Thermo Scientific). Absorbance values were read at 450 nm.

### 2.6. Detection of Oligomeric *α*-Syn

Oligomeric *α*-syn levels in RBCs were assessed using an immunoenzymatic assay, as previously described [46, 51–53] using an *α*-syn biotinylated antibody [53]. The wells were coated with the mouse monoclonal *α*-syn 211 antibody (sc-12767, Santa Cruz Biotechnology) and incubated with RBCs (0.04 mg/100 *μ*l) for 2 h. Streptavidin-horseradish peroxidase conjugate antibody (1 : 1000, GE Healthcare) was used for antigen detection of the biotinylated antibody. After three washes with PBS-T, TMB was added in each sample, as reported above.

### 2.7. Statistical Analysis

Data are expressed as the mean value ± SD. A normal distribution for age was found for the subjects included in this study. Differences between groups (i.e., young versus older, ATHL versus SED) were evaluated by one-way ANOVA followed by a Kruskal-Wallis post hoc test. *P* values were adjusted with Sidak's multiple comparison test. Such analyses were confirmed by a two-way ANOVA test. Correlation between variables was determined by simple linear regression analysis, whereas covariate analysis was performed by partial correlation matrix. All statistical procedures were performed using the StatView programme (Abacus Concepts Inc., SAS Institute, Cary, NC) [46, 54].

## 3. Results

### 3.1. Research Plan and Measured Parameters

The present study explored whether, in healthy individuals, physical activity or age is related to different parameters evaluated at the peripheral level, including the CpG site methylation within *SNCA* intron 1 (*SNCA_I1_*); the concentration of the protein encoded by *SNCA* (*α*-syn), considering total levels and the contribution of its oligomeric form; and the levels of the DNA methyltransferase enzymes of maintenance (Dnmt1) and *ex novo* (Dnmt3a).

Healthy subjects (*n* = 84, sedentary and athletes) were enrolled in the study. The calculation of mean values and correlation analyses of the parameters related to *SNCA* epigenetic modification were initially performed in the entire population. Then, statistical analyses of such parameters (in terms of mean value comparisons and correlation analyses) were conducted in samples derived from the stratification of the same initial entire population in the following subgroups: (1) sedentary (SED, *n* = 52) and athlete (ATHL, *n* = 32) subgroups, when the parameter for subdivision was the degree of physical activity; (2) “young” (*n* = 50) and “older” (*n* = 34) subgroups, when the parameter for subdivision was age.

### 3.2. Descriptive Statistics

A descriptive table reporting age, body mass index (BMI), heart rate, and physical activity level (15-level Borg's scale) of the entire population and of each group is shown (Table 1). Young and older groups presented a mean age of 34.6 ± 8.6 and 58.8 ± 7.2, respectively, and did not present differences in sex and BMI (*P* = 0.2702). ATHL and SED did not present differences in age (*P* = 0.1586) and BMI (*P* = 0.0746). As expected, the level of physical exercise was significantly higher in the ATHL group than in the SED group (*P* < 0.001).

### 3.3. Entire Population Stratified in Subgroups: Mean Values and Comparison of the Measured Parameters

#### 3.3.1. *SNCA_I1_* Relative DNA Methylation

The levels of *SNCA* CpG island relative methylation were measured in DNA samples extracted from the blood of all subjects. Specifically, a CpG island region within *SNCA_I1_*, previously demonstrated as a transcriptionally active region [35], was considered. The analysed CpG islands were from 89,836,281 to 89,836,520 of chromosome 4 (NC_000004.12). Herein, the percentage of *SNCA_I1_* relative DNA methylation of the total population was 4.37 ± 3.07 (Table 2).

#### 3.3.2. Dnmt1 and Dnmt3a Expression Levels

Dnmt1 is the maintenance methylation enzyme, which preserves the methylation patterns established early in development [55]. By contrast, Dnmt3a has been shown to methylate hemimethylated and unmethylated DNA with equal efficiencies *in vitro* [56]. Both Dnmts are expressed not only in the adult brain [57] but also in the hematopoietic cells [58] and constitute candidate targets to study the variation of DNA methylation with ageing and physical exercise.

**Figure 1:**
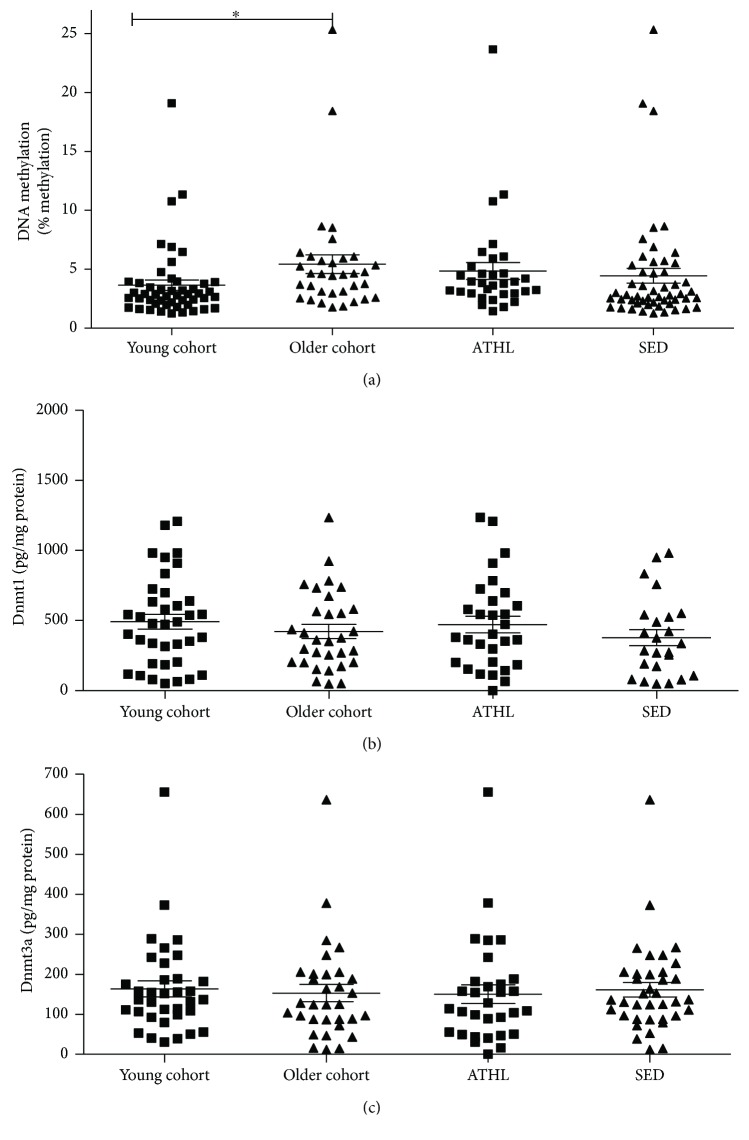
(a) *SNCA_I1_* relative DNA methylation levels were evaluated by MRSE digestion of genomic DNA extracted from the blood cells of healthy subjects and followed by quantitative real-time PCR. The results were expressed as the percentage of methylation in young, older, ATHL, and SED subgroups. (b) Dnmt1 and (c) Dnmt3a levels were determined in the blood of young, older, ATHL, and SED subgroups. Differences between groups (i.e., young versus older and ATHL versus SED) were evaluated by one-way ANOVA followed by a Kruskal-Wallis post hoc test. *P* values were adjusted with Sidak's multiple comparison test. ^∗^*P* < 0.05 between the indicated subgroups.

In the entire population, the Dnmt1 and Dnmt3a expression level was 490 ± 372 and 158 ± 119 pg/mg protein, respectively (Table 2). ELISA assays did not show significant differences in Dnmt1 protein levels in the different subgroups (older versus young subjects: *P* = 0.4832; ATHL versus SED: *P* = 0.2292; Figure 1(b)). Similar results were obtained for Dnmt3a protein concentration (older versus young subjects: *P* = 0.8369; ATHL versus SED: *P* = 0.7012; Figure 1(c)). Overall, these data suggest that the observed differences in the *SNCA* methylation status between young and older subjects are not related to changes in Dnmt1-3a levels.

#### 3.3.3. *α*-Syn Concentrations

Total and oligomeric concentrations of the gene product, *α*-syn, were determined in RBCs isolated from the entire population (Table 2). RBCs were chosen among blood cells because they contain approximately 98% of the total amount of circulating *α*-syn [43]. Total *α*-syn and oligomeric *α*-syn mean values were 62.5 ± 52.3 and 11.0 ± 5.4 ng/mg protein, respectively (Table 2).

**Figure 2:**
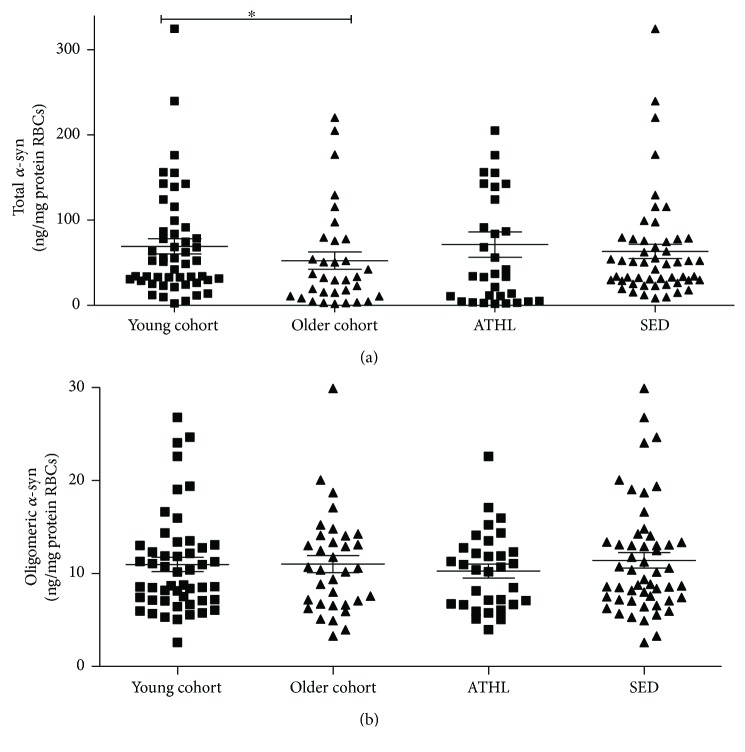
Determination of total and oligomeric *α*-syn in RBCs. Total (a) and oligomeric (b) *α*-syn levels were determined in RBCs from the young, older, ATHL, and SED subgroups, as described in Methods. Differences between groups (i.e., young versus older and ATHL versus SED) were evaluated by one-way ANOVA followed by a Kruskal-Wallis post hoc test. *P* values were adjusted with Sidak's multiple comparison test. ^∗^*P* < 0.05 between the indicated subgroups.

As depicted in Figure 2(a), older subjects displayed significant lower *α*-syn concentrations compared to young subjects (*P* = 0.0436), suggesting that *α*-syn may decrease with age in RBCs. By contrast, ATHL and SED presented comparable concentrations of the protein (Figure 2(a)), suggesting that physical exercise poorly modulated the RBC pool of *α*-syn.

Due to the high standard deviation of total *α*-syn values (see Table 2), the frequency distribution of this parameter was analysed. The distributions of values led us to select a population presenting RBC *α*-syn concentrations of 76.3 ng/mg protein or lower (confidence interval (CI) = 95%), on which some correlation analyses were performed (see below).

As depicted in Figure 2(b), oligomeric *α*-syn in RBCs did not significantly differ between young and older subjects (*P* > 0.999), as well as between ATHL and SED (*P* = 0.3603).

### 3.4. Correlation of the Measured Parameters with Age

**Figure 3:**
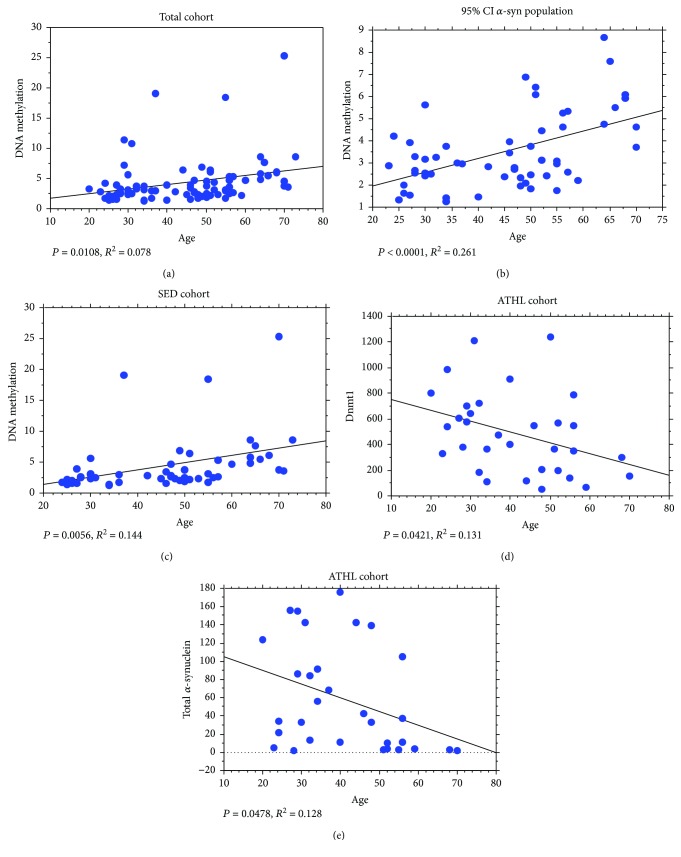
Correlation analyses between *SNCA_I1_* relative DNA methylation levels, Dnmt1, total *α*-syn, and age. Correlation analysis between *SNCA_I1_* relative DNA methylation and age (a) in the total population, (b) in the “95% CI *α*-syn population” (i.e., subjects characterized by RBC *α*-syn concentration of 76 ng/mg protein or lower), and (c) in the SED group. (d) Correlation analysis between Dnmt1 levels and age in the ATHL group. (e) Correlation analysis between total *α*-syn levels in RBCs and age in the ATHL group. The correlation between variables was determined by simple linear regression analysis. *P* and *R*^2^ were reported in the respective panels.

*SNCA_I1_* methylation at the CpG site presented a positive correlation with age for blood sampling in the total population (Figure 3(a)), consistent with the data in Figure 1(a). The negative correlation between *SNCA* methylation and age was particularly stronger in subjects presenting RBC *α*-syn concentrations of 76 ng/mg protein or lower (Figure 3(b)). Interestingly, such a correlation was found in the SED subgroup (Figure 3(c)) but not in the ATHL (*P* = 0.7608). These data suggest that ATHL may present additional factors other than age that contribute to the level of intron1-*SNCA* methylation.

For Dnmt levels, a weak inverse correlation between Dnmt1 expression and age was observed in the ATHL subgroup (Figure 3(d)). No other significant relationship with age was found either for Dnmt1 (total population: *P* = 0.1659; young: *P* = 0.8194; older: *P* = 0.3235; and SED: *P* = 0.6095) or Dnmt3a (total population: *P* = 0.5384; young: *P* = 0.9284; older: *P* = 0.3760; ATHL: *P* = 0.3632; and SED: *P* = 0.8969) concentrations.

Consistent with the data obtained for Dnmt1, total *α*-syn concentrations in RBCs showed an inverse correlation with age of blood sampling in the ATHL subgroup only (Figure 3(e); young: *P* = 0.2972; older: *P* = 0.8112; and SED: *P* = 0.6089).

Finally, no statistical significance was observed between age for blood sampling and oligomeric *α*-syn levels (total population: *P* = 0.6054; young: *P* = 0.4513; older: *P* = 0.6769; ATHL: *P* = 0.1884; and SED: *P* = 0.2824).

### 3.5. Correlation of the Measured Parameters with the Level of Physical Activity

**Figure 4:**
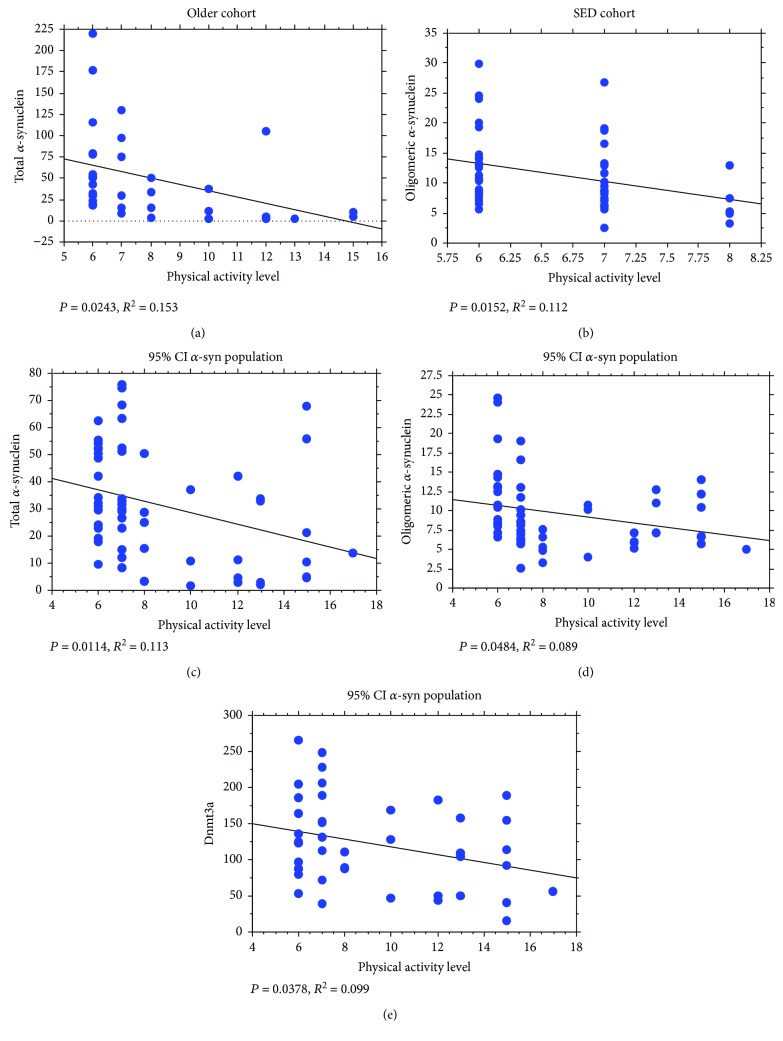
Correlation between the levels of total and oligomeric *α*-syn in RBCs and the level of physical activity. (a) Correlation analysis between total *α*-syn levels and physical activity in the older group. (b) Correlation analysis between oligomeric *α*-syn levels and physical activity in the SED group. Correlation analysis between (c) total *α*-syn, (d) oligomeric *α*-syn levels and (e) Dnmt3a and physical activity in the “95% CI *α*-syn population.” Correlation between variables was determined by simple linear regression analysis. *P* and *R*^2^ were reported in the respective panels.

The two-way ANOVA analysis suggests significant differences in the *SNCA_I1_* methylation rate between ATHL and SED groups (*P* < 0.0001). Nevertheless, the level of physical activity did not show any significant correlation with *SNCA_I1_* methylation rate in any of the analysed groups (young: *P* = 0.3668; older: *P* = 0.4685; ATHL: *P* = 0.6768; and SED: *P* = 0.8815). Similarly, the level of physical exercise did not correlate either with Dnmt1 (total population: *P* = 0.8956; young: *P* = 0.6999; older: *P* = 0.8015; ATHL: *P* = 0.6377; and SED: *P* = 0.6047) or Dnmt3a (total population: *P* = 0.9652; female: *P* = 0.3622; male: *P* = 0.3087; young: *P* = 0.9309; older: *P* = 0.5777; ATHL: *P* = 0.3721; and SED: *P* = 0.2811) expression. Interestingly, total *α*-syn concentrations in RBCs inversely correlated with the rate of physical activity in the older subjects (Figure 4(a)). In contrast, the oligomeric form of *α*-syn showed an inverse correlation with the physical activity score in the SED subgroup only (Figure 4(b)). Moreover, the two-way ANOVA analysis evidenced significant differences in oligomeric *α*-syn content between the ATHL and SED groups (*P* = 0.0308).

When the population presenting RBC *α*-syn concentrations ≤ 76 ng/mg protein was selected, the negative correlations with level of physical exercise were evidenced not only for total *α*-syn (Figure 4(c)) but also for oligomeric *α*-syn (Figure 4(d)) and Dnmt3a levels (Figure 4(e)).

### 3.6. Correlation of *SNCA_I1_* Relative DNA Methylation with Dnmt Levels

**Figure 5:**
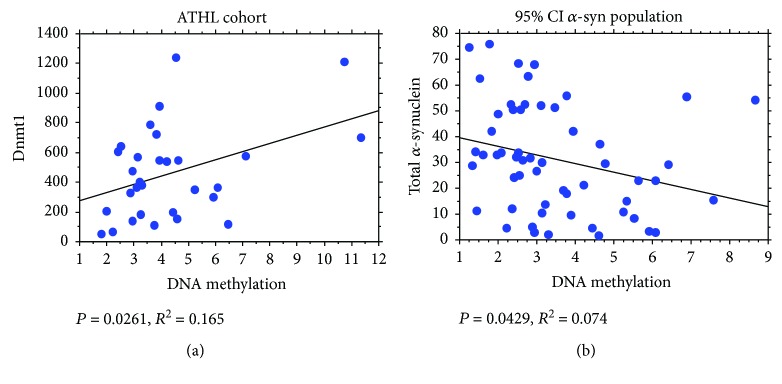
Correlation between Dnmt1 and total *α*-syn and DNA methylation. (a) Correlation analysis between Dnmt1 and DNA methylation levels in the ATHL subgroup. (b) Correlation analysis between total *α*-syn and DNA methylation levels in the “95% CI *α*-syn population.” Correlation between variables was determined by simple linear regression analysis. *P* and *R*^2^ were reported in the respective panels.

*SNCA_I1_* methylation levels correlated directly with blood Dnmt1 expression in ATHL (Figure 5(a)). No significant correlations were found in the other subgroups for Dnmt1 (young: *P* = 0.4420; older: *P* = 0.3531; and SED: *P* = 0.2232).

The expression of Dnmt3a did not correlate with the *SNCA* methylation rate in any of the analysed groups (young: *P* = 0.8902; older: *P* = 0.3932; ATHL: *P* = 0.5221; and SED: *P* = 0.2778), thus confirming that blood Dnmt3a poorly modulates intron1-*SNCA* methylation.

### 3.7. Correlation of *SNCA_I1_* Relative DNA Methylation with *α*-syn Concentrations

Interestingly, in subjects presenting RBC *α*-syn concentrations ≤ 76 ng/mg protein, a significant correlation between intron1-*SNCA* methylation rates and total *α*-syn levels was evidenced (Figure 5(b)). These data suggest a causal relationship between such an epigenetic modification and production of the protein encoded by *SNCA_I1_* up to this range of *α*-syn.

By contrast, the *SNCA_I1_* methylation degree was not related to the RBC content of *α*-syn (total population: *P* = 0.9486; young: *P* = 0.2944; older: *P* = 0.6740; ATHL: *P* = 0.1221; and SED: *P* = 0.5875). Similar results were obtained for the oligomeric form of *α*-syn (total population: *P* = 0.4930; young: *P* = 0.2916; older: *P* = 0.9736; ATHL: *P* = 0.4990; and SED: *P* = 0.6521). These data indicate the lack of a direct relationship between the expression of these enzymes and the levels of *SNCA* protein product.

### 3.8. Correlation of Dnmt Levels with *α*-Syn Concentrations

**Figure 6:**
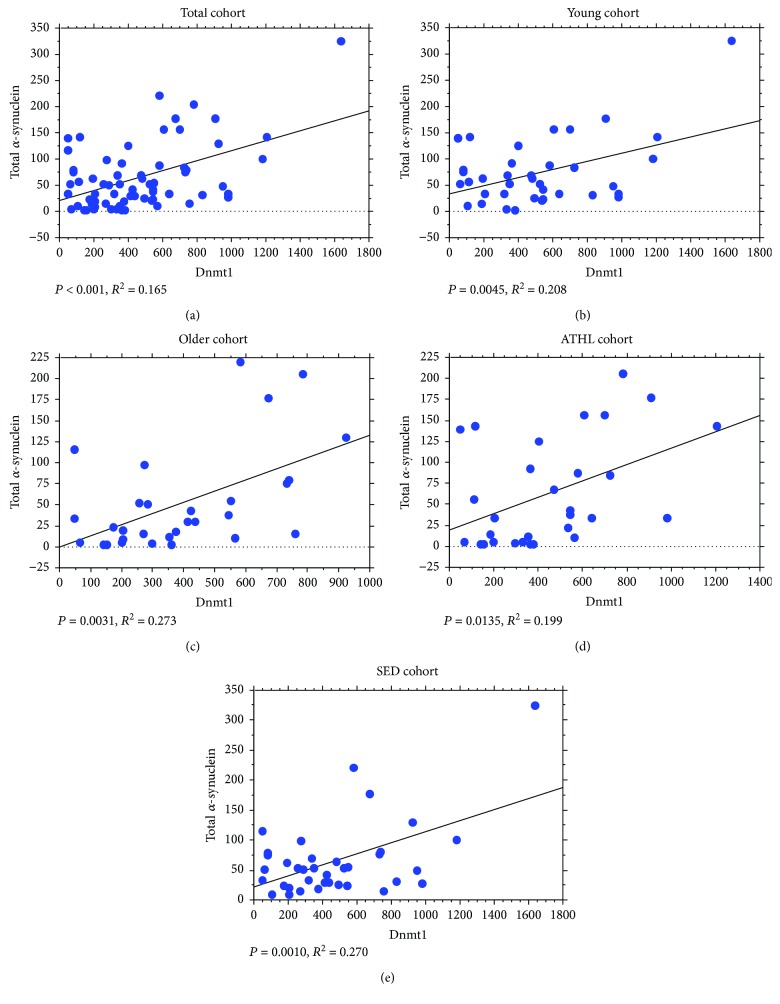
Correlation between levels of total *α*-syn in RBCs and Dnmt1. Correlation analysis between total *α*-syn levels and Dnmt1 in the total population (a), the young group (b), the older group (c), the ATHL group (d), and the SED group (e). Correlation between variables was determined by simple linear regression analysis. *P* and *R*^2^ were reported in the respective panels.

Furthermore, the relationship between Dnmt levels and *α*-syn concentrations was observed. Surprisingly, a strong direct correlation between Dnmt1 expression and total *α*-syn levels in RBCs was found in the total population (Figure 6(a)). Such a direct association remained significant in all the subgroups (Figures 6(b)–6(e)).

**Figure 7:**
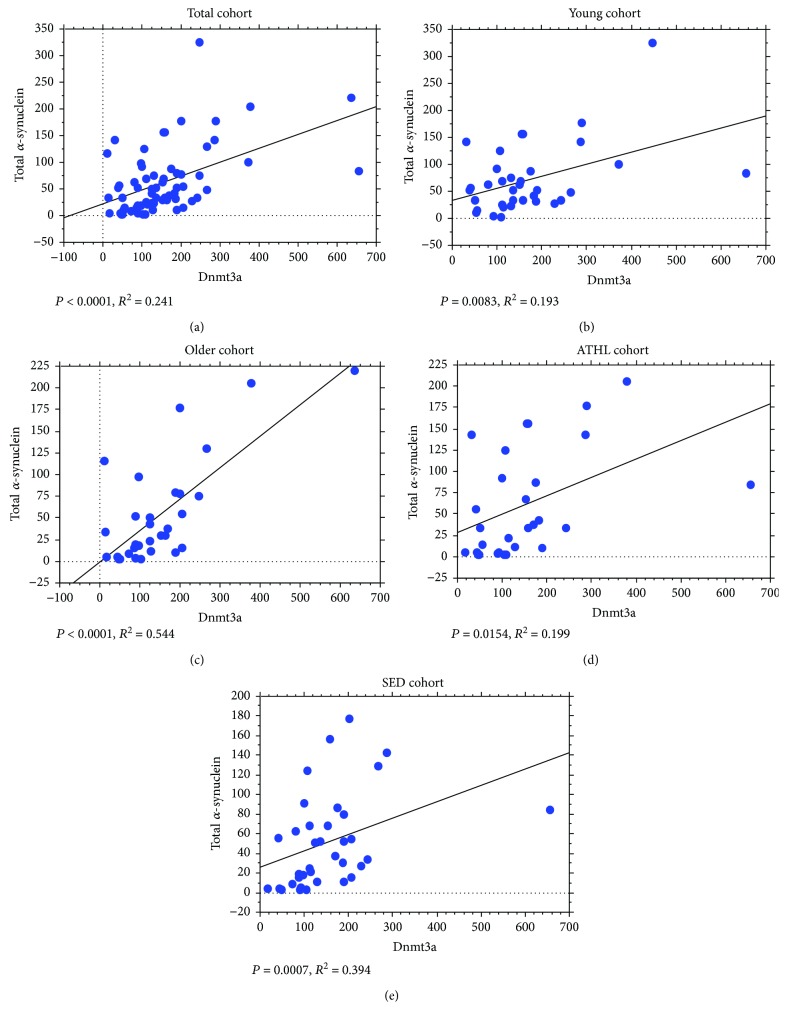
Correlation between levels of total *α*-syn in RBCs and Dnmt3a. Correlation analysis between oligomeric *α*-syn levels and Dnmt3a in the total population (a), the young group (b), the older group (c), the ATHL group (d), and the SED group (e). Correlation between variables was determined by simple linear regression analysis. *P* and *R*^2^ were reported in the respective panels.

Similarly, the Dnmt3a content in the blood directly correlated with the total *α*-syn levels in RBCs in the total population (Figure 7(a)), as well as in all the subgroups (Figures 7(b)–7(e)).

**Figure 8:**
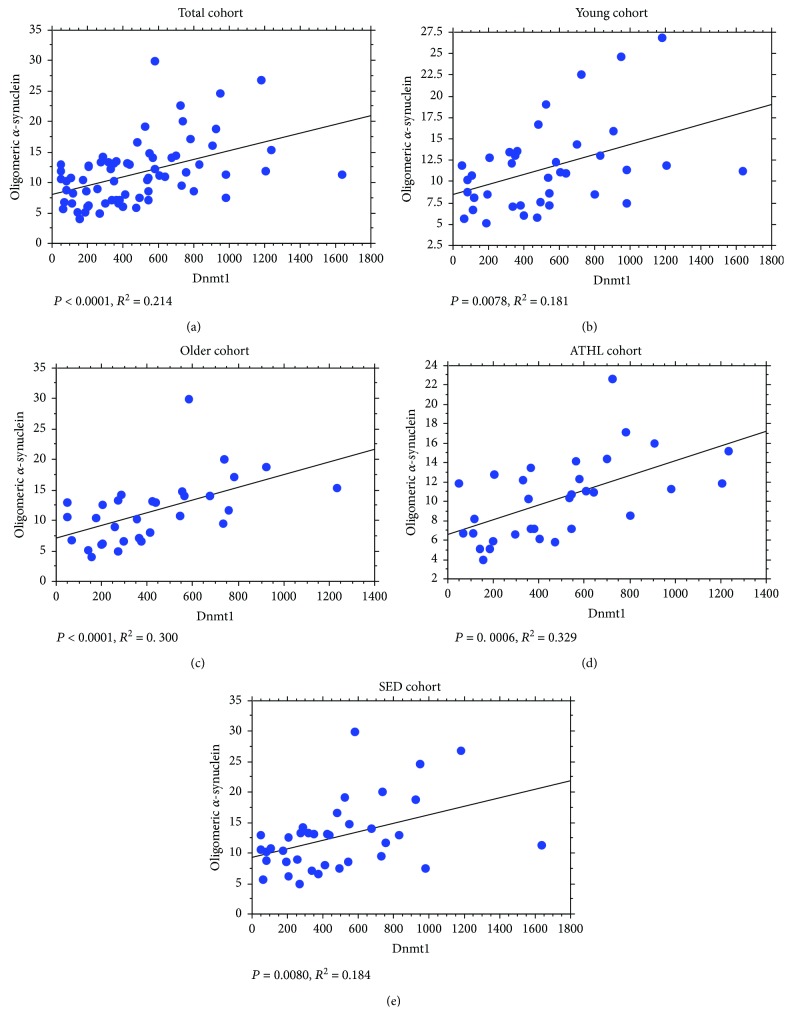
Correlation between levels of oligomeric *α*-syn in RBCs and Dnmt1. Correlation analysis between oligomeric *α*-syn levels and Dnmt1 in the total population (a), the young group (b), the older group (c), the ATHL group (d), and the SED group (e). Correlation between variables was determined by simple linear regression analysis. *P* and *R*^2^ were reported in the respective panels.

**Figure 9:**
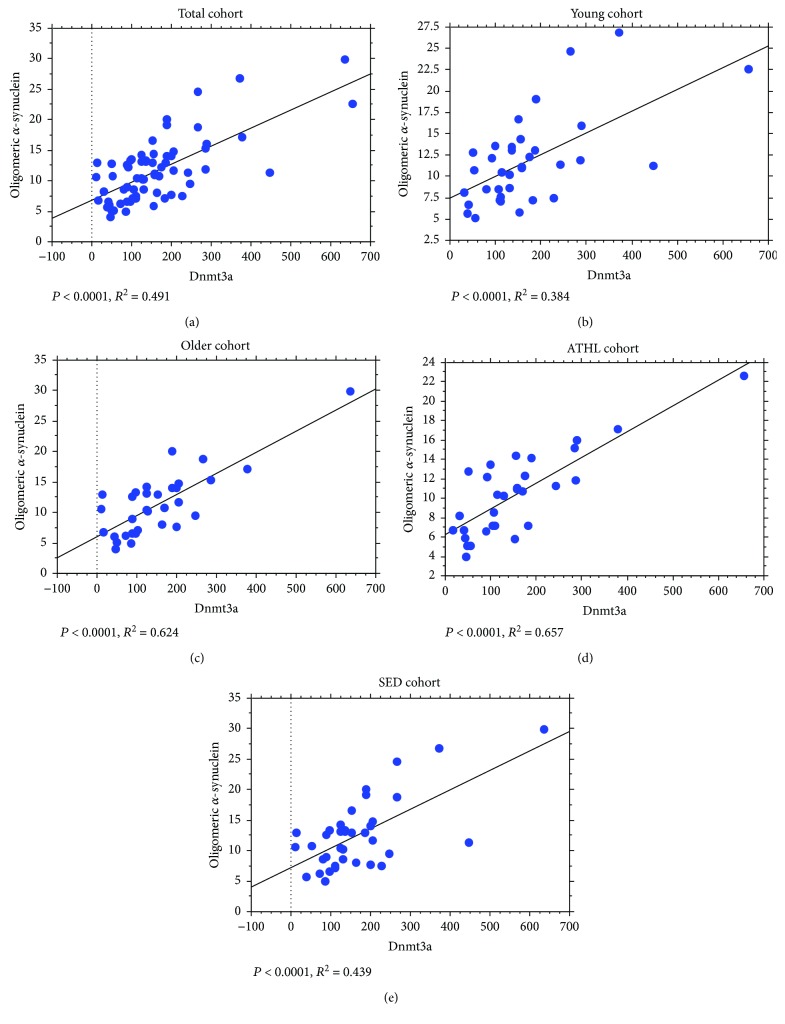
Correlation between levels of oligomeric *α*-syn in RBCs and Dnmt3a. Correlation analysis between oligomeric *α*-syn levels and Dnmt3a in the total population (a), the young group (b), the older group (c), the ATHL group (d), and the SED group (e). Correlation between variables was determined by simple linear regression analysis. *P* and *R*^2^ were reported in the respective panels.

The abovementioned positive correlations between Dnmt1/Dnmt3a levels and total *α*-syn concentrations were maintained for its oligomeric form, in all the analysed subgroups (Dnmt1 in Figures 8(a)–8(e)); Dnmt3a in Figures 9(a)–9(e).

## 4. Discussion

Here, the influence of age and physical activity on the epigenetic modification of the *α*-syn gene (*SNCA*) was explored. The major findings of this paper are as follows: (i) DNA methylation of intron1-*SNCA* was directly correlated with age; (ii) total *α*-syn concentrations in RBCs were lower in the older subjects; (iii) Dnmt levels were directly correlated with both total and oligomeric *α*-syn; (iv) in the 95-percentile population, the RBC levels of total *α*-syn levels were inversely related to the methylation status of intron1 *SNCA*; and (v) in the same selected population, the physical activity level was inversely related to the total and oligomeric *α*-syn levels in RBCs, as well as to Dnmt3a concentrations. Taken together, our data shed light on ageing- and physical exercise-induced changes on the methylation status and protein levels of *α*-syn, which accumulates as a misfolded oligomer in PD.

Epigenetic mechanisms and particularly DNA methylation were demonstrated to regulate brain ageing and age-related neurodegenerations. In particular, epigenetic regulation of the *α*-syn-encoding gene (*SNCA*) has been greatly explored focusing on the methylation status of intron1 CpG islands [35–37, 39–42, 50, 59]. Nevertheless, controversial findings have been reported in both brain and peripheral tissues of patients affected by PD [34, 36, 39–42].

If ageing remains the main contributing factor to PD pathogenesis, beneficial effects have been described for moderate physical activity [12, 21, 32], although the underlying regulatory mechanisms are not completely understood [60]. Alterations in gene expression as a consequence of physical training are frequently described. The mechanisms of the described alteration in gene expression mediated by physical activity could depend on epigenetic changes in global or gene-specific DNA methylation levels [12]. In this respect, a genome-wide analysis of DNA methylation recently highlighted the importance of epigenetic mechanisms in muscle adaptation to regular exercise [57, 60].

In the present study, healthy subjects (sedentary and athletes) were enrolled to investigate the influence of age and physical exercise on the methylation status of *SNCA*. This parameter was related to the blood content of total and oligomeric forms of *α*-syn protein, as well as to the blood concentrations of DNA methyltransferases. In particular, RBCs were chosen as a peripheral model because they accumulate misfolded proteins and are particularly susceptible to oxidative stress [47–49].

First, *SNCA* methylation levels were shown to be significantly higher in the older subgroup than in the young subgroup and to directly correlate with age. Similarly, DNA analysis from brain tissues recently revealed a slight increase of intron1-*SNCA* DNA methylation levels in presumably healthy individuals during ageing [61].

Next, *α*-syn protein accumulation was measured in RBCs. Older subjects presented lower levels of total but not oligomeric *α*-syn. Similar data on the protein levels were reported in RBCs, plasma, or platelets of healthy subjects or PD patients [62–66]. Moreover, a significant inverse correlation with age in the selected population presenting RBC *α*-syn concentrations up to 76.3 ng/mg protein (confidence interval = 95%) was observed. In the latter population, intron1-*SNCA* DNA methylation levels were inversely related to total *α*-syn concentrations. These data suggest that hypermethylation of intron1-*SNCA* can lead to a lower concentration of total *α*-syn in the blood, at least for a population presenting up to 76.3 ng/mg protein. In fact, the current hypothesis is that the minor levels presented by an elderly population or PD patients are the result of protein sequestration into oligomeric forms [62–66]. Further investigations are needed to clarify the effective influence of *SNCA* methylation on *α*-syn accumulation in the peripheral cells.

The link between *SNCA* methylation, *α*-syn concentrations, and Dnmts was then examined. Indeed, the decrease in global genomic methylation (hypomethylation) that occurs with ageing has been often proposed as a consequence of decreased Dnmt expression [9, 67–69]. However, contradictory evidence for both methylcytosine levels and Dnmt expression was shown during hippocampal ageing [7, 70–76]. Here, no significant differences in the expression of Dnmt1 or Dnmt3a were found between the analysed subgroups. By contrast, a reduction of nuclear Dnmt1 levels was demonstrated in brain samples from PD patients and *α*-syn transgenic mice, suggesting a mechanism by which *α*-syn may exclude the enzyme from the nucleus, resulting in further hypomethylation of *α*-syn CpG islands [59].

Surprisingly, both Dnmt1 and Dnmt3a levels directly correlated with the concentrations of total and oligomeric *α*-syn. These data suggest that Dnmt concentrations are not strictly related to *SNCA* methylation status in this group of healthy subjects. It is hypothesized that such Dnmt is highly involved in the methylation of genes different from *SNCA*.

For the role of physical exercise, sedentary and athletes were found to present similar percentages of *SNCA* methylation, *α*-syn concentrations, and Dnmt levels. Although it is well recognized that physical activity can control gene expression through epigenetic alterations [11], effective modulation of the methylation status of neurodegeneration-related genes remains to be largely investigated, particularly in peripheral tissues. Recently, exercise was shown to increase the global DNA methylation profile in the rat hypothalamus [77] or to induce DNA hypomethylation of the promoter IV-BDNF [18], as well as “at specific CpG site located within a VegfA promoter” [11, 78]. The latter findings were related to a significant reduction of Dnmt3b mRNA in the hippocampus of exercised rats, suggesting that genome-wide DNA hypomethylation can occur in the brain.

Surprisingly, when the population presenting up to 76.3 ng/mg protein of *α*-syn was selected, the level of physical exercise inversely correlated not only with total *α*-syn levels in RBCs but also with the oligomeric form and with Dnmt levels. These findings suggest that within the limits of *α*-syn accumulation, regular activity can modulate DNA methylation enzymes and reduce protein accumulation. In this respect, it should be mentioned that additional mechanisms on the regulation of *α*-syn expression cannot be excluded and will be investigated in future studies.

## 5. Conclusions

In conclusion, we show that DNA methylation of intron1-*SNCA* was modified with ageing but not with physical exercise. Within a moderate range of *α*-syn, RBC levels of the protein were inversely related to the methylation status of intron1-*SNCA* and the physical activity level.

Such findings shed light on the effects of physical exercise on ageing-associated alterations of epigenetic processes in peripheral cells.

## Figures and Tables

**Table 1:** Demographic and clinical analyses of the total population and of the subgroups. The data are the mean ± SD. BMI: body mass index; ATHL: athletes; SED: sedentary; W: women; M: men.

	*N*	Age (y)	BMI	Heart rate	15-level Borg's scale
Total population	84	44.4 ± 14.4	23.5 ± 2.0	57.6 ± 4.8	9.7 ± 3.8
SED	52 (M = 20; W = 32)	45.9 ± 14.3	24.2 ± 1.4	62.5 ± 5.1	6.6 ± 0.6
ATHL	32 (M = 20; W = 12)	41.4 ± 13.7	23.6 ± 1.6	50.2 ± 3.9	13.7 ± 2.0
Young subjects	50 (M = 22; W = 28)	34.6 ± 8.6	23.3 ± 1.9	55.8 ± 3.7	8.8 ± 3.2
Older subjects	34 (M = 18; W = 16)	58.8 ± 7.2	23.8 ± 2.2	59.4 ± 5.9	13.2 ± 2.2

**Table 2:** Values of total *α*-syn and oligomeric *α*-syn (ng/mg total protein), percentage of DNA methylation, and levels of Dnmt1 and Dnmt3a (pg/mg protein) in the indicated subgroups. The values are expressed as mean ± SD.

	Total *α*-syn	Oligomeric *α*-syn	DNA methylation	Dnmt1	Dnmt3a
Total population	62.5 ± 52.3	11.0 ± 5.4	4.37 ± 3.07	490 ± 372	158 ± 119
SED	63.2 ± 50.1	11.4 ± 5.9	4.45 ± 3.58	447 ± 295	161 ± 110
ATHL	58.6 ± 42.1	10.3 ± 4.2	4.03 ± 1.96	540 ± 407	155 ± 129
Young subjects	69.2 ± 40.8	11.0 ± 5.4	3.50 ± 3.03	505 ± 381	164 ± 117
Older subjects	52.4 ± 38.4	11.0 ± 5.0	5.42 ± 4.03	445 ± 355	156 ± 125

*SNCA_I1_* DNA methylation was significantly higher in the older group than in the young group (Figure 1(a), *P* = 0.0148), suggesting that age may influence epigenetic remodeling of the *α*-syn gene. By contrast, comparable levels in the methylation status of intron1-*SNCA* were found between ATHL and SED (Figure 1(a), *P* = 0.5442).
